# Sericin and swimming on histomorphometric parameters of denervated plantar muscle in Wistar rats

**DOI:** 10.1590/S1679-45082018AO4137

**Published:** 2018-04-19

**Authors:** André Junior Santana, Jean Carlos Debastiani, Pâmela Buratti, Ana Luiza Peretti, Regina Inês Kunz, Rose Meire Costa Brancalhão, Lucinéia de Fátima Chasko Ribeiro, Márcia Miranda Torrejais, Gladson Ricardo Flor Bertolini

**Affiliations:** 1 Universidade Estadual do Oeste do Paraná, Cascavel, PR, Brazil

**Keywords:** Peripheral nerves, Muscle fibers, skeletal, Exercise, Sciatic nerve/injuries, Proteins, Rats, Wistar, Nervos periféricos, Fibras musculares esqueléticas, Exercício, Nervo isquiático/lesões, Proteínas, Ratos Wistar

## Abstract

**Objective:**

To analyze the combined effects of the silk protein sericin and swimming exercise on histomorphometry of the plantar muscle in Wistar rats.

**Methods:**

Forty adult rats were randomly allocated into 5 groups comprising 8 animals each, as follows: Control, Injury, Sericin, Swim, and Swim plus Sericin. Three days after crushing of the sciatic nerve the rats in the Swim and Swim plus Sericin Groups were submitted to swimming exercise for 21 days. Rats were then euthanized and the plantar muscle harvested and processed.

**Results:**

Cross-sectional area, peripheral nuclei and muscle fiber counts, nucleus/fiber ratio and smallest muscle fiber width did not differ significantly between groups. Morphological analysis revealed hypertrophic fibers in the Swim Group and evident muscle damage in the Swim plus Sericin and Injury Groups. The percentage of intramuscular collagen was apparently maintained in the Swim Group compared to remaining groups.

**Conclusion:**

Combined treatment with sericin and swimming exercise did not improve muscle properties. However, physical exercise alone was effective in maintaining intramuscular connective tissue and preventing progression of deleterious effects of peripheral nerve injury.

## RESUMO

**Objetivo:**

Analisar o efeito da proteína sericina associada ao exercício físico de natação na histomorfometria do músculo plantar de ratos Wistar.

**Métodos:**

Foram utilizados 40 ratos adultos divididos aleatoriamente em 5 grupos, com 8 animais cada: Controle, Lesão, Sericina, Natação, Natação e Sericina. Três dias após a compressão do nervo isquiático, os Grupos Natação e Exercício e Sericina foram submetidos ao exercício físico de natação durante 21 dias. Após, os animais foram sacrificados, e o músculo plantar foi processado.

**Resultados:**

Não houve diferença da área da secção transversa entre os grupos, quantidade de núcleos periféricos, quantidade de fibra, relação núcleo/fibra e diâmetro menor. A análise morfológica revelou que no Grupo Natação ocorreu hipertrofia das fibras, assim como nos Grupos Exercício e Sericina e Lesão, o dano muscular foi evidente. O percentual de conjuntivo intramuscular parece ter sido mantido no Grupo Exercício em relação aos demais grupos.

**Conclusão:**

A associação da proteína sericina e exercício físico de natação não foi eficiente na melhora das propriedades musculares, embora a aplicação do exercício físico tenha sido eficiente na manutenção do conjuntivo intramuscular, e no não agravamento dos efeitos deletérios consequentes da lesão nervosa periférica.

## INTRODUCTION

Peripheral nerve injuries (PNI) may result in partial or total sensory, autonomous and motor function loss, leading to tissue retraction, pain and edema, among many other secondary complications.^(^ ^1^ ^)^ In developed countries, estimated PNI incidence ranges from 13 to 23 cases per 100 thousand inhabitants/year, particularly among the economically active population (*i.e.*, young people aged 21 to 30 years).^(^ ^2^ ^)^

The sciatic nerve of rats is widely used in experimental nerve crushing models due to its morphological features, ease of surgical access and availability of previous data for comparison.^(^ ^3^ ^)^ Nerve crushing leads to pelvic limb muscle weakness^(^ ^4^ ^)^ and denervation; atrophy, fibrosis and transient dilation of the intramuscular capillary bed then ensue, with resulting increased blood and extracellular fluid volume, and proteolysis.^(^ ^5^ ^)^

The connective tissue, a carbohydrate- and proteinrich extracellular matrix, plays a significant role in muscle fiber morphological and functional integrity.^(^ ^6^ ^)^ This tissue has several functions: it fills in the space between muscle fibers and allows interfiber binding and alignment; coordinates the transmission of forces generated by muscles to tendons and bones to generate movement; provides support to nerves and blood vessels; in addition to lubricating structures to facilitate gliding.^(^ ^7^ ^)^

Skeletal muscle in known to regenerate fast even after severe damage, a function of neuromuscular plasticity.^(^ ^8^ ^)^ The peripheral nerve system shares this high recovery capacity; however, functional outcomes are often poor, particularly due to the potentially long distance between compromised axons and target organs, which may preclude reconnection.^(^ ^2^ ^)^

Swimming exercise improves muscle properties via aerobic training, and is one of the therapeutic strategies employed in rehabilitation.^(^ ^9^ ^)^ The benefits of physical exercise for muscle regeneration, atrophy prevention and improvement of muscle structural properties have been demonstrated. However, effects on skeletal muscle following sciatic nerve crush injury are debatable, particularly where exercise type, intensity and timely introduction are concerned.^(^ ^10^ ^,^ ^11^ ^)^

Just as physical exercise, biocompatible materials have a role to play. Among other biocompatible materials, silkworm cocoon proteins fibroin and sericin have been investigated in several health science fields.^(^ ^12^ ^)^ Some studies suggest that sericin peptides combined with physical exercise boost aerobic performance, fat oxidation and testosterone levels.^(^ ^13^ ^,^ ^14^ ^)^ The biocompatibility of sericin for treatment of burns and wounds has also been demonstrated.^(^ ^15^ ^)^ Still, in spite of promising biological features, the impact of sericin on neuromuscular regeneration and the effects of its combination with therapeutic strategies, such as swimming exercise, remain to be determined.

## OBJECTIVE

To analyze the combined effects of sericin and swimming on histomorphometry of the plantar muscle following sciatic nerve crushing in Wistar rats.

## METHODS

A total of 40 Wistar rats weighing 300±50g were used in this study. Rats were maintained in a 12:12 hour light: dark photoperiod at 24±1°C temperature; water and feed were offered *ad libitum.* Rats were randomly allocated to one of five experimental groups comprising eight animals each, as follows: Control (Ct), Injury (Inj), Sericin (Ser), Swim (Sw) and Sericin plus Swim (Ser+Sw). This study was approved by the Ethics Committee on Use of Animals, *Comitê de Ética no Uso de Animais* (CEUA) of *Universidade Estadual do Oeste do Paraná*, Carvavel, Paraná.

### Sciatic compression experimental protocol

The nerve crush model in this study consisted of experimental sciatic nerve axonotmesis. Animals in Groups Inj, Ser, Sw and Ser+Sw were weighed and anesthetized via intraperitoneal injection of ketamine hydrochloride (95mg/kg, Dopalen, Brazil) and xylazine (12mg/kg, Anasedan, Brazil) prior to surgery. Following consciousness level assessment (absence of motor response to tail and interdigital skin fold clamping), rats were positioned in ventral recumbent position with thoracic and pelvic limbs in abduction, and the mid-third of the right thigh clipped.

An incision parallel to the biceps femoris muscle fibers was then created to expose the sciatic nerve. The nerve was crushed for 30 seconds using hemostatic forceps; crushing pressure was standardized by closing the ratchet on the second catch.^(^ ^16^ ^)^ Finally, the nerve was repositioned, the skin closed with monofilament nylon in a simple interrupted fashion and polyvinylpyrrolidoneiodine (Povidine^®^) applied to the surgical wound. Rats were kept under the above-mentioned conditions after surgery.

### Resistance swimming exercise protocol

The swimming exercise protocol was adapted from Bertolini et al.,^(^ ^17^ ^)^ Only Groups Sw and Ser+Sw were submitted to swimming; however, the rats in the remaining groups were also exposed to water for 10 seconds over the course of the treatment, so that all groups were submitted to similar aquatic environment derived stress. Rats were gradually adapted and trained to swim for 15 days prior to initiation of the nerve crushing protocol. Swimming treatment was started on the third postoperative day. Swimming exercise was performed in a 60cm deep oval-shaped tank with 200-liter capacity; water level was maintained at 40cm and water temperature at 32°C throughout.

Rats were always weighed prior to exercise sessions for determination of weight overloads (10% body weight applied via a lead shot-filled velcro waist belt). The 21-day treatment protocol consisted of Monday-through-Friday swimming sessions of progressive duration for 3 weeks, as follows: 15', 20’ and 25’ in the first, second and third postoperative week respectively.

### Morphological and morphometric analyses

Rats were weighed, anesthetized and decapitated in guillotine 24 hours after the last exercise session. The plantar muscle was then dissected away and the proximal segment processed for morphological analysis.^(^ ^18^ ^)^

For muscle fiber study, the specimens were kept at room temperature for 30 to 40 minutes and covered with powder for preservation, according to the technique described by Moline et al.,^(^ ^19^ ^)^ Specimens were then frozen in liquid nitrogen for 2 minutes, conditioned in cryotubes and stored in Biofreezer at −80°C for later processing. Frozen muscle segments were transferred to a cryostat chamber (Lupetec CM2850 Cryostat microtome) at −20°C and let to sit for 30 minutes for temperature stabilization. Semi-serial 7*μ*m cross-sections were then obtained, dehydrated, diaffanized, mounted onto slides using Permount (Thermo Fisher Scientific^®^, New Jersey, USA) and stained with hematoxylin and eosin (general muscle tissue analysis) or Masson's trichrome (connective tissue analysis).

Slides were analyzed under light microscope (BX60 Olympus^®^, Tokyo, Japan). Ten 40x magnification images were randomly selected for measurement of cross-sectional area and smallest muscle fiber width, with ten fibers measured per image using Image-Pro Plus 6.0 (Media Cybernetics^®^, Silver Spring, USA), *i.e*, one-hundred measurements per animal.

Connective tissue density, muscle fiber and peripheral nuclei counts were determined from ten randomly selected 40x magnification images (ten measurements per animal).

Endomysial and perimysial connective tissue density were analyzed using GNU Image Manipulation Program - GIMP 2.0 (GNU General Public License^®^, Berkeley, California, USA). Relative connective tissue area (density area) was calculated as the total of pixels in microphotographs divided by the total of pixels in connective tissue markings.

Muscle fibers and nuclei were morphologically identified, marked and counted. Nucleus/fiber ratio was estimated by dividing total nuclei counts by total muscle fiber counts within a single field. Fibers and peripheral nuclei projected over the upper and left borders of each microphotograph were excluded to prevent sampling errors.

### Statistical analysis

Data were analyzed using BioEstat^®^ 5.0 and expressed as means and standard deviations. One-way Analysis of Variance (ANOVA) and the post t-test (LSD) were used. The level of significance was set at 5%.

## RESULTS

### Histomorphometric analyses of the plantar muscle

Histomorphometric analyses of the plantar muscle (*i.e.*, cross-sectional area, muscle fiber and peripheral nuclei counts, nucleus/fiber ratio, smallest muscle fiber width and connective tissue density in the endomysium and perimysium) are presented in table 1. Cross-sectional area, muscle fiber counts and smallest muscle fiber width differed significantly between the Ct and remaining groups, with no differences between the latter. Nuclei counts and nucleus/fiber ratios did not differ significantly between groups. Intramuscular connective tissue was significantly increased in Groups Inj, Ser and Ser+Sw compared to the Ct Group (156%, 67% and 58%, respectively), and differed significantly between the Inj and treated groups (Ser, Sw and Ser+Sw; 53%, 115% and 62% increase respectively). The Ser Group also differed significantly from the Sw Group, with 40% increase in intramuscular connective tissue.

**Table 1:** Measurements of muscle fiber area

Parameters	Groups				
	Control	Lesion	Sericin	Swim	Sericin+Swim
Area	2,740.9±254.0^a^	1,533.7±279.9^b^	1,584.5±220.6^b^	1,498.3±181.9^b^	1,452.5±341.2^b^
Diameter menor	43.6±1.3^a^	33.4±3.6^b^	34.7±3.0^b^	33.1±1.6^b^	32.3±4.6^b^
Number of fiber	343.2±52.9^a^	522.7±48.3^b^	634.2±187.6^b^	551.8±82.5^b^	555.2±120.0^b^
Number of fiber	364.8 ±95.6^a^	609.5±191.2^a^	526.2±117.4^a^	693.0±224.5^a^	679.4±243.3^a^
Nucleus/fiber	1.1±0.3^a^	1.1±0.3^a^	0.9±0.3^a^	1.2±0.2^a^	1.3±0.7^a^
Connective %	2.6±0.6^a^	6.7±1.1^b^	4.3±1.0^cd^	3.1±0.8^ae^	4.1±1.5^de^

Similar letters indicate statistical similarity. Different letters indicate statistically different data among the groups (p<0.05). Values expressed as mean±standard deviation.

Results show that physical exercise was not able to induce improvements or changes in muscle properties in the Inj Group in this study, but had no negative impacts and was effective in maintaining intramuscular connective tissue when used alone, as compared to remaining treatment protocols. Sericin (Ser) or sericin combined with swimming (Ser+Sw) were not effective therapeutic modalities when compared to swimming alone Sw Group.

### Morphological analysis of the plantar muscle

Morphological analysis of the plantar muscle in the Ct Group revealed normal fibers with preserved polygonal shape, peripheral myonuclei in subsarcolemal position close to the cell membrane and preserved fascicular pattern (connective tissue arranged in bundles), with no apparent changes in the perimysium or endomysium, except for the presence of blood capillaries.

In the Inj Group, plantar muscle damage resulting from denervation translated into fibers with irregular contour, fascicular disorganization with increased intramuscular collagen, loss of polygonal shape (polymorphic fibers), reduced fiber size, atrophy and enlarged peripheral nuclei (not statistically significant). Muscle fibers with reduced area, polymorphism and atrophic fibers were noted in the Ser Group, along with thicker connective tissue, enlarged and centrally positioned peripheral myonuclei, and larger numbers of blood capillaries.

**Figure 1:**
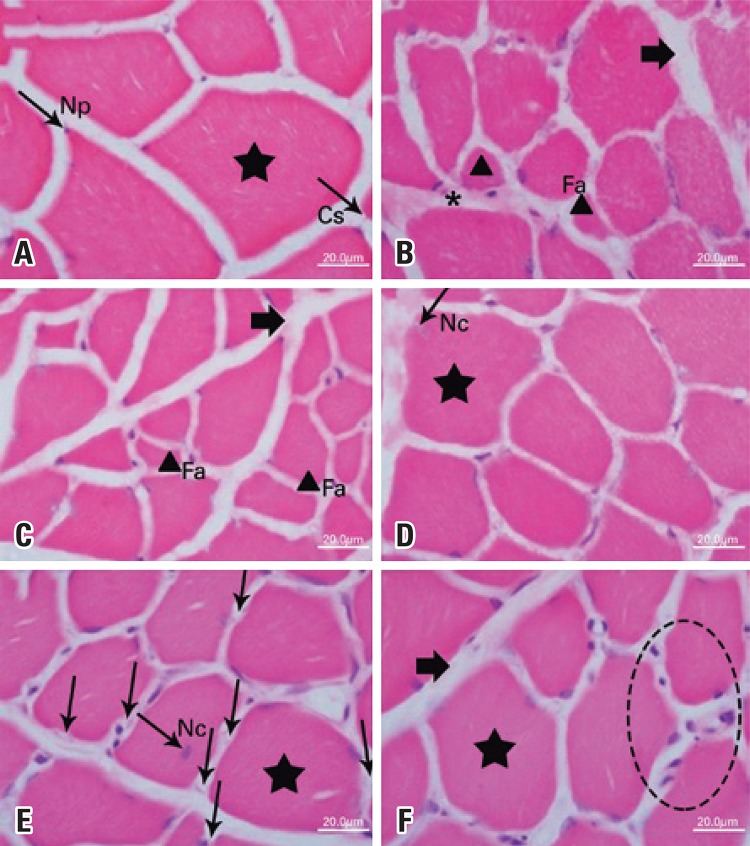
Microphotographs of cross-sectional views of the plantar muscle of Wistar rats, hematoxylin & eosin stain. Control Group (A), Injury Group (B), Sericin Group (C), Swim Group (D) and Sericin+Swim Group (E and F). (A) Note polygonal shaped muscle fibers (star), peripheral nuclei (Np) and blood capillaries (Cs). (B) Note atrophic muscle fibers (triangle), increased connective tissue density (large arrow) and fibroblasts (*). (C) Note atrophic (triangle) and polymorphic fibers. (D) Note hypertrophic muscle fibers (star) and centrally located nucleus (Nc). (E) Note hypertrophic fibers (star), centrally located nucleus (Nc) and increased number of blood capillaries (vertical arrows). (F) Note increased numbers of peripheral nuclei (dotted ellipse), hypertrophic fiber (star) and connective tissue thickening (large arrow)

In the Sw Group, some hypertrophic muscle fibers were noted and peripheral myonuclei and centralized nuclei counts appeared increased, with many nuclei surrounded by basophilic haloes; tissue organization was normal and myoblasts were present at the injury site. The plantar muscle in the Ser+Sw Group had slightly disorganized connective tissue and enlarged peripheral myonuclei. Fibers were polygonal shaped and some were hypertrophic; central nuclei and surrounding basophilic haloes were also noted (Figure 1).

## DISCUSSION

The sciatic nerve crushing model employed in this study reproduces axonotmesis type injuries^(^ ^16^ ^)^ characterized by interruption of neuromuscular stimuli. This was reflected in morphological changes typical of denervation related muscle damage observed in the plantar muscle of Group Inj animals, such as polymorphic fibers of reduced width, increased connective tissue density and presence of central nuclei. These findings support those of Malysz et al.,^(^ ^11^ ^)^ who described structural changes and skeletal muscle atrophy following axonotmesis type injury to the sciatic nerve. Rosa Jr. et al.,^(^ ^20^ ^)^ also reported changes in skeletal muscle fibers of the flexus digitorum longus and soleus muscles in response to sciatic nerve injury. According to Cavalcante et al.,^(^ ^21^ ^)^such morphological changes lead to decrease in muscle mass, fiber width and force generation, and ultimately to organ function compromise.

Analyses in this study were carried out 22 days following nerve injury; therefore, signs of muscle regeneration could be observed, such as centralized nuclei and fibroblast clumps suggestive of protein synthesis^(^ ^22^ ^)^ which indicate an attempt at overall tissue regeneration in response to organic injury. Similar responses were observed by Tanaka et al.,^(^ ^10^ ^)^ in a study reporting spontaneous recovery of the soleus muscle, six weeks after tibial nerve injury.

Satisfactory results of therapeutic physical exercise aimed at maintenance or improvement of muscle morphology following PNI have been reported in literature.^(^ ^10^ ^,^ ^11^ ^)^ Physical exercise promotes muscle hypertrophy^(^ ^9^ ^)^ and contributes to atrophy prevention.^(^ ^10^ ^)^Swimming exercise was selected because it combines aerobic activity with cardiorespiratory overload, given water viscosity implies resistance to movement in all directions.^(^ ^23^ ^)^ However, our findings regarding recovery from muscle atrophy secondary to sciatic nerve injury did not support treatments employed in the Sw and Ser+Sw Groups. The training protocol selected (resistance with overload limited to 10% of body weight) was likely insufficient to generate muscle mass increases capable of mitigating muscle atrophy. Artifon et al.,^(^ ^24^ ^)^ also failed to demonstrate changes in skeletal muscle morphometric parameters of rats submitted to sciatica followed by a progressive swimming exercise protocol similar to the one employed in this study, with the exception of overload.

In this study, swimming exercise had no negative impacts on degenerative changes resulting from nerve crushing. This may have reflected lower impacts and stress on structures and muscle fibers generated by swimming compared to ground exercise - a result of properties such as buoyancy, viscosity and water temperature, or overload and total duration of training.

Tissue remodeling is mediated by several cytokines and growth factors involved in fibrogenesis regulation in healing of wounds, muscle lacerations and denervation injuries, such as transforming growth factor beta 1 (TGF-β1), matrix metalloproteinases)MMPs) and tissue inhibiting metalloproteinases (TIMPS). Ozawa et al.,^(^ ^25^ ^)^ described changes in these regulating factors in the initial phase)days 3, 7 and 14) of skeletal muscle remodeling in rats submitted to complete sciatic nerve denervation. Better understanding of mechanisms underlying PNI related neuromuscular changes is paramount for proper therapeutic intervention aimed at functional recovery of the neuromuscular system.

The connective tissue is responsible for morphological and functional integrity of skeletal muscles, which are able to adapt in response to stimuli such as denervation, among others.^(^ ^26^ ^,^ ^27^ ^)^ Intramuscular connective tissue increase was observed in this study, with significant differences (156%, 67% and 58%, respectively) between the Inj, Ser and Ser+Sw Groups compared to the Ct Group, and between the Inj and remaining treated groups (Ser, Sw and Ser+Sw; 53%, 115% and 62% increase, respectively). Significant differences were also observed between the Ser and Sw Groups, with 40% connective tissue increase in the first. Increased intramuscular collagen in the gastrocnemius muscle following sciatic nerve injury was reported by Salonen et al.,^(^ ^26^ ^)^ while Ozawa et al.,^(^ ^25^ ^)^ observed increases in skeletal muscle collagen following complete sciatic denervation, with 80% increase in collagen expression on day 14 post-injury.

According to Minamoto,^(^ ^28^ ^)^ skeletal muscle denervation may cause connective tissue density to increase, as this tissue responds to injury with fibroblast proliferation and synthesis of extracellular matrix components. Type III collagen synthesis increases during cell repair, particularly in the perimysium and endomysium. Increased connective tissue density area reflects type I collagen proliferation in the endomysium and perimysium and, given its reduced elasticity, may lead to decreased muscle elasticity, with potential muscle function compromise.^(^ ^26^ ^,^ ^28^ ^)^

The fact that intramuscular connective tissue was maintained in the group treated with swimming in this study shows that physical exercise, although insufficient for overall muscle morphology recovery, was able to prevent progression of deleterious PNI effects on muscles. The opposite was documented in the group treated with sericin, in which increased connective tissue density was observed. Combined treatment with swimming exercise and sericin did not improve outcomes.

The association of sericin and swimming exercise in the acute phase of recovery was not an effective therapeutic modality for treatment of PNI in this study. However, physical exercise alone was effective in maintaining connective tissue proportions. Health care professionals must be aware of the close relationship between the nervous and musculoskeletal systems, as well as of pertaining clinical applicability. Therapeutic protocols must contemplate not only muscles and nerves, but also periarticular structures involved in nerve injuries, so as to accelerate the regeneration process with views to restoring patient function.

## CONCLUSION

Sciatic nerve axonotmesis was able to promote muscular injury effects 22 days following injury, and combined treatment with sericin and swimming exercise did not accelerate muscle recovery. However, swimming exercise was effective in maintaining intramuscular connective tissue density. Therefore, the need for therapeutic physical exercise following compressive nerve injury cannot be overemphasized, if tissue properties required for effective repair are to be maintained.
